# Prognostic Implication of SOX2 Expression Associated with p16 in Oropharyngeal Cancer: A Study of Consecutive Tissue Microarrays and TCGA

**DOI:** 10.3390/biology9110387

**Published:** 2020-11-09

**Authors:** Jungirl Seok, Chang Hwan Ryu, Junsun Ryu, Ji-Hyun Kim, Sang-Jin Lee, Weon Seo Park, Yuh-Seog Jung

**Affiliations:** 1 Department of Otorhinolaryngology-Head and Neck Surgery, National Cancer Center, 323 Ilsan-ro, Ilsandong-gu, Goyang-si 10408, Gyeonggi-do, Korea; junn279@gmail.com (J.S.); changhwanr@ncc.re.kr (C.H.R.); jsryu@ncc.re.kr (J.R.); jihyun6342@ncc.re.kr (J.-H.K.); 2 Division of Cancer Immunology, National Cancer Center, 323 Ilsan-ro, Ilsandong-gu, Goyang-si 10408, Gyeonggi-do, Korea; leesj@ncc.re.kr; 3 Department of Pathology, National Cancer Center, 323 Ilsan-ro, Ilsandong-gu, Goyang-si 10408, Gyeonggi-do, Korea; thymus@ncc.re.kr

**Keywords:** oropharyngeal cancer, human papillomavirus, SOX2, TCGA, immunohistochemistry

## Abstract

**Simple Summary:**

The role of human papillomavirus (HPV) in oropharyngeal cancer (OPSCC) as a cause agent has been reported in much of the literature. As a surrogate marker, p16 immunohistochemical staining is used as the standard for classifying OPSCC, and the prognosis of p16+ OPSCC has been reported to be better than p16− OPSCC. However, it was necessary to study what is the next biomarker that could predict the prognosis after classification by p16. We assumed that SOX2 may be a potential biomarker. For each p16+ and p16− OPSCC, SOX2 was used to analyze whether the degree of expression level differed in survival and recurrence rates. The results showed that both immunohistochemical staining and mRNA expression level of SOX2 significantly affected the survival and recurrence rates of p16+ OPSCC patients in two different datasets which were constructed in differently ways. Our study presented the clinical applicability of SOX2 as a biomarker.

**Abstract:**

For oropharyngeal squamous cell carcinoma (OPSCC), there are not enough additional robust biomarkers for subgrouping after the distinct classification using p16. As SOX2 is an emerging biomarker for cancer treatment, its clinical implication in OPSCC was evaluated using a consecutive tissue microarray (TMA) cohort consisting of 111 patients who underwent surgery as an initial treatment from May 2002 to December 2016 and 79 patients in The Cancer Genome Atlas (TCGA) dataset. In both datasets, p16+*/SOX2*^High^ (HPV+/*SOX2*^High^ in TCGA) showed the best prognosis among the four groups classified by SOX2 and p16 for 5-year overall survival (OS) and recurrence (all *p* < 0.05), but SOX2 did not make a significant difference in the prognosis of the p16− group. In the TMA cohort, SOX2^High^ was significantly correlated with response to radiotherapy and lower pathologic T classification in the p16+ group (*p* = 0.001). In TCGA, correlations between *SOX2* and tumor stage classification or radiotherapy were not observed; however, HPV+/*SOX2*^High^ had a significantly low tumor mutation burden among the four groups (all *p* < 0.05). In summary, SOX2 was proven to be a potential marker to predict overall survival and recurrence in p16+ OPSCC. However, the role of SOX2 has not yet been confirmed in p16− OPSCC patients.

## 1. Introduction

Head and neck cancers, which occur at the oral cavity, oropharynx, nasopharynx, hypopharynx and unspecified pharynx, are the seventh most frequent cancer and the ninth leading cause of cancer-related death worldwide [1]. The majority of cases represent head and neck squamous cell carcinoma (HNSCC) that arises from the oral cavity, oropharynx and larynx, which accounts for more than 90% of head and neck cancers [2]. The prognosis of HNSCC is still poor even, with combined multimodality treatment, including surgery, radiotherapy and chemotherapy [3]. The current classification based on anatomic site and tumor stage fails to reflect due to their remarkable heterogeneity [4], and the lack of improving patient survival from the uniformity of treatment propelled the establishment of a novel strategy that uses biomarkers based on the genetic and biological behavior of tumors [3,5].

SOX2, a pluripotency-associated transcription factor, is an emerging biomarker for the prediction of prognosis and therapeutic targets for cancer treatment because it plays an important role in the development and maintenance of the stem cell state and is associated with cancer progression [6]. However, whether higher SOX2 expression is a favorable or unfavorable risk factor depends on the primary organ of cancer [7,8,9,10,11,12]. Even in head and neck cancer, SOX2 and its associated pathway have been presented as novel biomarkers but the results are controversial [3,13,14,15,16,17,18,19].

Among head and neck cancers, oropharyngeal squamous cell carcinoma (OPSCC) is one of the major subsites of HNSCC. Since human papilloma virus (HPV) is known to be a causative agent [20], OPSCC is now classified into HPV+ and HPV− OPSCC by using immunohistochemistry (IHC) of p16^INK4a^, which is a surrogate marker for HPV status [21]. HPV-related OPSCC is recognized as a distinct category because of its unique etiology, biology, clinical presentation and therapeutic responses [2]. Recently, two groups have different staging systems in the latest guidelines [22]. However, there are not enough additional robust biomarkers for subgrouping after molecular classification using p16 IHC.

In light of evidence that SOX2 can be a biomarker, we hypothesized that SOX2 may be a potential marker for OPSCC and sought to find the implication of SOX2 in OPSCC after classification by p16^INK4a^ IHC. After evaluation of the hypothesis using a head and neck cancer cohort of our institute, we also examined whether a similar finding can be validated at the mRNA expression level through The Cancer Genome Atlas Head–Neck Squamous Cell Carcinoma (TCGA-HNSC) dataset.

## 2. Materials and Methods

### 2.1. Ethical Consideration

All participants provided written informed consent according to the policies and procedures approved by the institutional review board of the National Cancer Center, Republic of Korea (approval number: NCC-NCS-08200).

### 2.2. Patients

From May 2002 to December 2016, a “Head and Neck Cancer Tissue Microarray Cohort (TMA cohort)” was established for a total of 397 consecutive patients (431 specimens) who visited the head and neck cancer center of our institute and agreed to a prospective study. Inclusion criteria were adults over 18 years old who were diagnosed with head and neck cancer through surgery or biopsy, and cases of follow-up-loss before specimen acquisition were excluded from the cohort.

TMA blocks were generated using three representative tumor areas (2 mm in core size) and paired normal control tissue from formalin-fixed, paraffin-embedded tumor material and marked on standard hematoxylin/eosin (H&E)-stained sections for the expression of tissue markers [23].

The source (primary site, lymph node, etc.) and timing of tumor tissue sampling (surgery or biopsy), age at the time of tissue acquisition, clinical information related to radiation therapy and chemotherapy, cancer staging and the date of death and recurrence were collected. A total of 124 patients with oropharyngeal cancer were extracted from the cohort, including only the tissue at the primary site and excluding cases who received treatment other than surgery as an initial treatment.

**Figure 1:**
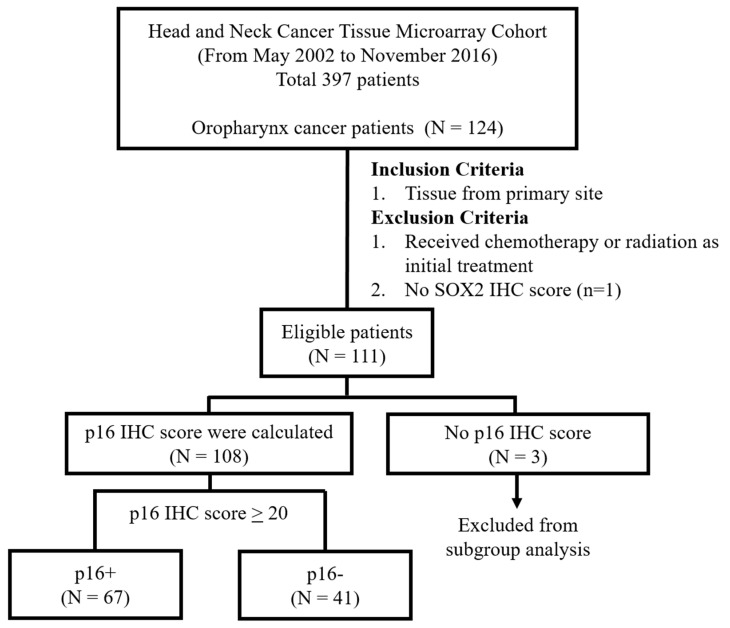
Schematic diagram of the study design.

A case without SOX2 IHC score was excluded, and a total of 111 eligible patients were enrolled (Figure 1). Radiotherapy as an adjuvant treatment for initial operation (postoperative radiotherapy; PORT) was defined when it was delivered within 180 days after initial surgery.

### 2.3. Immunohistochemistry of p16 and SOX2

**Figure 2:**
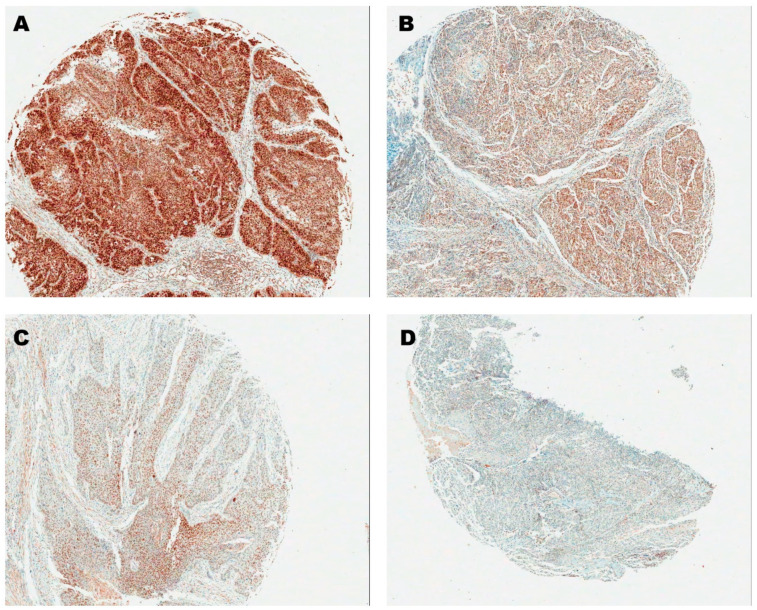
Exemplary images of SOX2 immunohistochemistry staining in tonsil tissue and their corresponding scores ((**A**): 30, (**B**): 20, (**C**): 10 and (**D**): 0).

Immunostaining of p16 protein was performed using a monoclonal anti-p16 antibody (clone E6h4; ready-to-use; Dako, Carpinteria, CA, USA), and that of SOX2 protein was performed using a rabbit polyclonal anti-SOX2 antibody (clone EPR3131; 1:100 dilution; Abcam, Australia). The method of scoring the IHC stating was similar to Park et al.’s previous research [24]: without clinical information of the samples, semiquantitative composite scoring was conducted by an experienced pathologist (W.S.P). The stained tumor was divided into 10 parts and each part was graded from 0 (negative) to 3 (strong). Accordingly, IHC scores from 0 to 30 were obtained (Figure 2). The score distribution of the two markers is shown in Figure S1. For p16 IHC, there were no scores between 11 and 27 and 25 was set as a cutoff value to define samples with a strong intensity as p16+.

### 2.4. The Cancer Genome Atlas (TCGA)

Clinical data and mRNA expression values obtained from tumor tissue were collected from the TCGA-HNSC dataset for 79 patients with OPSCC who had information on HPV status. HPV status was classified based on the item labeled “patient.hpv_test_results.hpv_test_result.hpv_status”. Information on survival and recurrence was collected based on the items labeled “followup_treatment_success”, “vital_status”, “primary_therapy_outcome_sucess” and “new_tumor_event_after_initial_treatment”, and the duration (days) of each status from the initial treatment was obtained from the corresponding items labeled “days_to_last_followup”, “days_to_death” and “days_to_new_tumor_event_after_initial_treatment”. TNM classification was defined based on the clinical information provided. Pathologic classification (pTNM) was adopted, but clinical classification (cTNM) was used when pTNM information was not provided or if there was a record of chemotherapy but no record of surgery. Information on radiotherapy as an adjuvant treatment was collected on the items labeled “radioation_therapy” and “postoperative_rx_tx”, only “days_to_radiation_therapy_start” was the same or less than 180 days following initial diagnosis was accepted.

### 2.5. Statistical Analysis

In the cohort, follow-up of 17 of the 111 eligible patients was lost at some point, and the information about recurrence was not tracked after, but the time of death was obtained from the Ministry of Public Administration, Republic of Korea. For these patients, 5-year overall survival (OS) was calculated based on the time of death, and 5-year recurrence was calculated based on the information at the last follow-up.

In the TCGA dataset, the expression level of *SOX2* mRNA was changed to a base-2 log scale and then standardized using the ‘scale’ function of R software. The immune cell signature was calculated using the single-sample gene set enrichment analysis (ssGSEA) method in the Gene Set Variation Analysis (GSVA) package of R software [25]. The tumor mutation burden (TMB) score was calculated as follows [26]:

Total number of truncating mutations × 1.5 + total number of nontruncating mutations × 1.0

In both datasets, the survival and recurrence rates were calculated using the Kaplan–Meier method. Hazard ratios (HRs) and confidence intervals (CIs) were calculated using the Cox regression method. The statistical significance of differences between the actuarial curves was tested by log-rank tests. A pairwise log-rank test was used as a post hoc analysis for comparison of more than two groups.

All statistical analyses were performed using R Statistical Software (version 3.5.1; R Foundation for Statistical Computing, Vienna, Austria). Time-dependent receiver operating characteristic (ROC) curve and area under curve (AUC) were estimated using the *survivalROC* package of R software [27].

## 3. Results

The cohort showed significant differences in age at operation, smoking, subsite, pathological T (pT) classification and postoperative radiotherapy and recurrence according to p16+ and p16− (all *p* < 0.01) (Table 1).

### 3.1. SOX2 Cut-Off Value and Correlation with p16

Using a time-dependent ROC curve, an SOX2 IHC score > 20 was determined as a cut-off value for 5-year overall survival in which the overall number of false positives and false negatives were minimized (AUC: 57.9, sensitivity: 43.9%, specificity: 74.0%). There was no IHC score between 25 and 20; accordingly, a group with an SOX2 IHC score of 25 or higher was referred to SOX2^High,^ and a group with 20 or lower was referred to SOX2^Low^.

SOX2^High^ showed a higher ratio in p16+ oropharyngeal cancer than p16−, but there was no statistically significant difference (76.1% vs. 63.4%, respectively, *p* = 0.157, Table 1). Age and sex were not significantly different in the two groups according to SOX2 (SOX2^High^ vs. SOX2^Low^, 61.6 ± 11.4 vs. 58.7 ± 10.5, *p* = 0.231 for age and 96.8% vs. 83.8% for fraction of male patient, *p* = 0.107 from Fisher’s exact test).

### 3.2. SOX2 and Pathological Classification

In this study, p16+ oropharyngeal cancer had a significantly higher proportion of low pathologic T classification (pT1 and -2) than the p16− group (91.0% vs. 68.3%, *p* = 0.003) and a higher proportion of pathologically identified lymph node metastasis without significance (pN+, 70.1% vs. 48.8% of pN−, *p* = 0.060).

**Figure 3:**
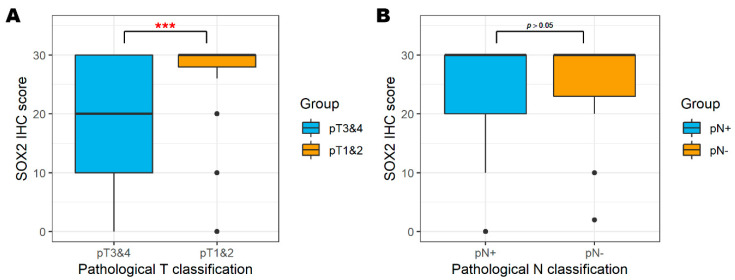
SOX2 IHC scores according to pathological T classification (**A**) and pathological N classification (**B**). *** *p* < 0.001.

Higher SOX2 status had more pT1 and -2 stage significantly; SOX2^High^ had 90% of pT1 and -2 and SOX2^Low^ had 68.3% (*p* = 0.003). In comparison with the SOX2 IHC score, lower scores were more distributed in higher pT classification (median [IQR] IHC score of pT3 and -4 vs. pT1 and -2:30 [28,29,30] vs. 20 [10,11,12,13,14,15,16,17,18,19,20,21,22,23,24,25,26,27,28,29,30], *p* < 0.001) (Figure 3). This trend was also significant in p16+ oropharyngeal cancer (proportion of SOX2^High^ in pT1 and -2 vs. pT3 and -4: 81.8% vs. 16.7%, *p* = 0.001, Fisher’s exact test) but not significant in the p16− group (proportion of SOX2^High^ in pT1 and -2 vs. pT3 and -4: 71.4% vs. 46.2%, *p* = 0.169, Fisher’s exact test) (Table 2). However, pN classification was not significantly different in either group (pN+, 60% in SOX2^High^ vs. 64.5% in SOX2^Low^, *p* = 0.661).

### 3.3. Risk Factors for Overall Survival and Recurrence

In the risk factor analysis (Table 3), a low SOX2 IHC score was identified as a significant risk factor for 5-year OS (HR (95% CI): 2.39 (1.19–4.81), *p* = 0.015) and 5-year recurrence (HR (95% CI): 2.72 (1.32–5.59), *p* = 0.005) in the univariate analysis. Accordingly, SOX2^High^ and SOX2^Low^ showed a significant difference in 5-year OS and recurrence (Figure S2). When SOX2 was multivariate analyzed with p16 and PORT, SOX2^Low^ was a still risk factor for 5-year recurrence (HR (95% CI): 2.33 (1.11–4.89), *p* = 0.025), but in 5-year OS, SOX2 was not significant but still showed a tendency to increase the risk (HR (95% CI): 1.99 (0.99–4.04), *p* = 0.054). Only p16 was a significant factor in both 5-year OS and recurrence in multivariate analysis.

**Figure 4:**
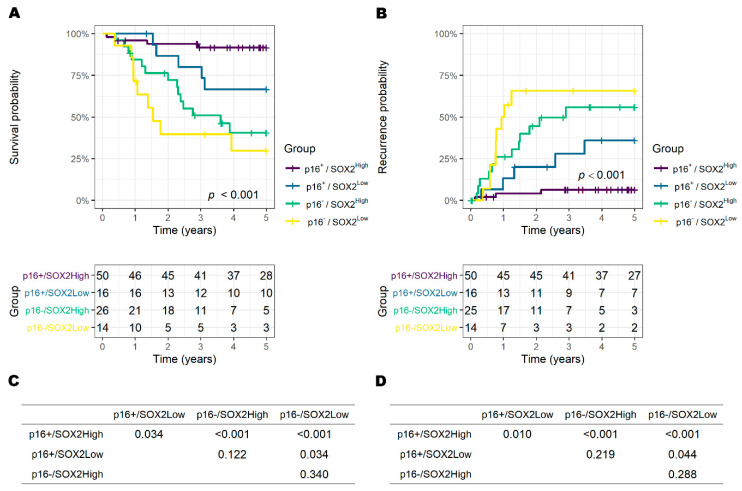
Survival and recurrence in four groups classified by p16+/− and SOX2^High/Low^. (**A**) The 5-year overall survival (OS) and number at risk table. (**B**) The 5-year recurrence and number at risk table. Pairwise log-rank test as a post hoc analysis for 5-year OS (**C**) and recurrence (**D**).

When p16 was included in the classification (Figure 4), the overall survival rate of p16+/SOX2^High^ was 91.7% (95% CI: 84.3–99.8), p16+/SOX2^Low^ was 66.7% (95% CI: 46.6–95.3), p16−/SOX2^High^ was 40.5% (95% CI: 24.4–67.3) and p16−/SOX2^Low^ was 29.8% (95% CI: 12.4–71.6). The 5-year recurrence rate of p16+/SOX2^High^ was 6.3% (95% CI: 0.0–1.3), p16+/SOX2^Low^ was 36.0% (95% CI: 4.5–57.1), p16−/SOX2^High^ was 55.9% (95% CI: 27.8–73.1) and p16− /SOX2^Low^ was 65.7% (95% CI: 27.6–83.8).

In a post hoc pairwise analysis of both 5-year OS and recurrence, p16+/SOX2^High^ was significant compared to p16+/SOX2^Low^, p16−/SOX2^High^ and p16−/SOX2^Low^, and p16+/SOX2^Low^ was significant compared to p16−/SOX2^Low^ (all *p* < 0.05). In the p16− group, p16−/SOX2^High^ showed a significantly better OS rate at 2 years after surgery (76.4% (95% CI: 61.5–94.9) vs. 39.7% (95% CI: 20.3–77.7), *p* = 0.043), but SOX2 did not make a significant difference from 3 years of observation.

When analyzing the type of recurrence, the TMA cohort had a total of 22 locoregional recurrence cases and 9 distant metastases as the first progression. At the time the recurrence was diagnosed, local recurrence and distant metastasis were detected simultaneously in four patients. There was significantly more locoregional recurrence in p16− than p16+ (16 (39.0%) vs. 6 (9.0%), *p* < 0.001) and more distant metastasis as the first progression, but this difference was not significant (6 (14.6%) vs. 3 (4.5%), *p* = 0.064). SOX2Low had significantly more locoregional recurrence (11 (35.5%) vs. 11 (14.3%), *p* = 0.013) but not distant metastasis (4 (12.9%) vs. 5 (6.5%), *p* = 0.276). Risk factor analysis revealed that p16 significantly increased the risk of both locoregional recurrence and distant metastasis (all *p* < 0.05), whereas SOX2Low significantly increased only locoregional recurrence, even when analyzed in combination with p16 (Table S1).

### 3.4. SOX2 and Radiotherapy

**Figure 5:**
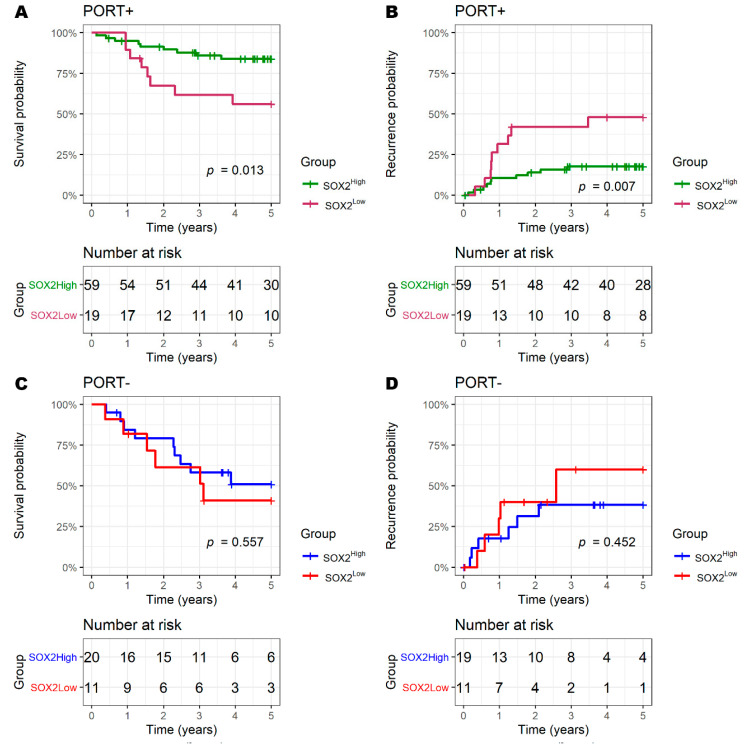
Prognosis according to SOX2 score and postoperative radiotherapy (PORT). In the PORT+ group, the expression level of SOX2 had a significant effect on survival (**A**) and recurrence (**B**). However, in the PORT- group, it did not significantly affect the survival (**C**) and recurrence (**D**).

The significance of SOX2 was different in patients according to PORT (Figure 5). The prognosis of the SOX2^High^ and SOX2^Low^ groups was significantly different in PORT+ patients (5-year OS of SOX2^High^ vs. SOX2^Low^: 83.8% (95%CI: 74.7–94.1) vs. 56.1% (95%CI: 37.4–84.4), *p =* 0.013; 5-year recurrence: 17.6% (95%CI: 7.1–27.0) vs. 47.9% (95%CI: 19.5–66.3), *p* = 0.007). Meanwhile, the difference in prognosis was not significant in PORT− patients (5-year OS of SOX2^High^ vs. SOX2^Low^: 50.8% (95%CI: 32.0–80.7) vs. 40.9% (95%CI: 19.4–86.3), *p* = 0.557; 5-year recurrence: 38.2% (95%CI: 8.4–58.3) vs. 60.0% (95%CI: 0.0–84.5), *p* = 0.452).

The difference in prognosis did not reach a significance level when divided by p16; however, the SOX2^High^ group tended to have an improved prognosis (5-year recurrence of SOX2^High^ vs. SOX2^Low^ in PORT+/p16+: 7.3% (95%CI: 0.0–14.8) vs. 25.9% (95%CI: 0.0–47.4), *p* = 0.088; 5-year OS in PORT+/p16−: 54.5% (95%CI: 32.8–90.4) vs. 14.3% (95%CI: 2.3–87.7), *p* = 0.065; 5-year recurrence in PORT+/p16−: 52.4% (95%CI: 15.1–73.3) vs. 85.7% (95%CI: 12.3–96.7), *p* = 0.092) (Figure S3).

### 3.5. Results of TCGA Data Set Analysis

In the TCGA-HNSC dataset, 79 of 528 cases were oropharyngeal cancer samples with HPV status based on in situ hybridization (ISH) or p16 tests. Accordingly, the cohort consisted of 54 cases (68.4%) of HPV+ and 25 cases (31.6%) of HPV− cancers; 54 cases (68.4%) had information about radiotherapy and 25 cases (31.6%) did not. Detailed demographic data of the TCGA subset are shown in Table S2.

The median value of standardized *SOX2* mRNA expression level was 0.35 [IQR: −0.17–0.71] for HPV+ and −0.59 [IQR: −1.31–0.38] for HPV−, significantly different (*p* = 0.005, Wilcoxon rank sum test). Regarding TNM classification, 0.03 [IQR: −0.61–0.38] for T3 and -4 and 0.38 [IQR: −0.36–0.72] for T1 and -2, which was a similar trend observed in the TMA cohort but not significant in the TCGA dataset (*p* = 0.141), 0.01 [−0.77–0.77] for patients without nodal metastasis (N−) and 0.15 [−0.35–0.63] for patients with node metastasis (N+), which was not significant (*p* = 0.664).

A cut-off value of −0.3020 was chosen using time-dependent ROC analysis to minimize false positives and false negatives, which was obtained from the overall survival data of the fourth year, which recorded the highest AUC (0.925). Accordingly, 55 cases were *SOX2*^High^ and 24 cases were *SOX2*^Low^.

*SOX2*^Low^ increased the risk of 5-year OS univariately (HR (95%CI): 8.98 (3.08–26.2), *p* < 0.001), even in multivariate analysis (HR (95%CI): 4.87 (1.44–16.5), vs. *SOX2*^High^ *p* = 0.011); rather, the significance of HPV− status was weakened in multivariate analysis (HR (95%CI): 3.24 (0.96–10.9), vs. HPV+, *p* = 0.059) when compared to univariate analysis (HR (95%CI): 7.11 (2.45–20.6), vs. HPV+, *p* < 0.001) (Table 4). For the 5-year recurrence, *SOX2*^Low^ was revealed to increase the risk only in univariate analysis (HR (95% CI): 2.82 (1.12–7.09), *p* = 0.028). Accordingly, 5-year OS and recurrence were significantly different between the *SOX2*^High^ and *SOX2*^Low^ groups (Figure S4).

When analyzed according to HPV status, *SOX2*^Low^ increased the risk of 5-year survival in HPV+ patients (HR (95% CI): 2.34 (1.66–65.6), *p* = 0.012 for HPV+ and 2.52 (0.68–9.35), *p* = 0.168 for HPV−), and a similar result was even seen when *SOX2* was set as a continuous value of standardized mRNA expression level (Table S3).

**Figure 6:**
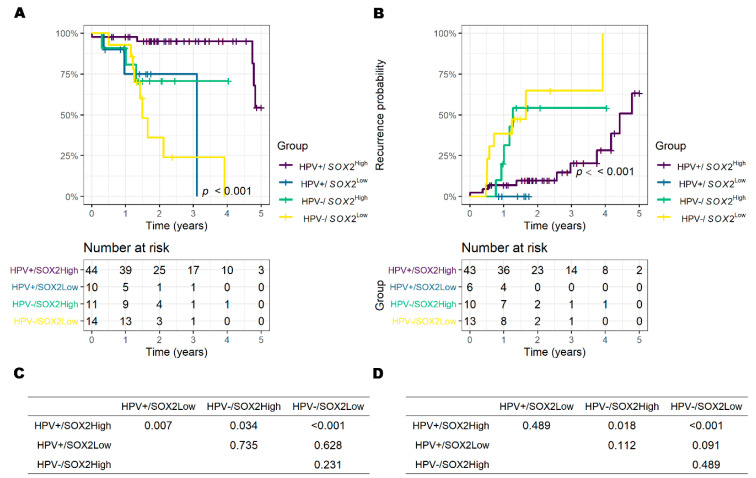
Survival and recurrence in four groups classified by HPV+/− and *SOX2*^High/Low^ in TCGA dataset. (**A**) The 5-year overall survival (OS) and number at risk table. (**B**) The 5-year recurrence and number at risk table. Pairwise log-rank test as a post hoc analysis for 5-year OS (**C**) and recurrence (**D**).

The overall survival and recurrence seemed to be significantly different between groups according to HPV status and *SOX2* mRNA expression (Figure 6). In the *post hoc* analysis, the 5-year overall survival of HPV+/*SOX2*^High^ was significantly improved compared with the other three groups (all *p* < 0.05), but there was no significant difference among HPV+/*SOX2*^Low^, HPV−/*SOX2*^High^ and HPV−/*SOX2*^Low^. For 5-year recurrence, HPV+/*SOX2*^High^ had the best prognosis, but only significant when compared to HPV−/*SOX2*^High^ and HPV−/*SOX2*^Low^ (all *p* < 0.05).

In contrast to the TMA cohort, RT was not a significant factor that affected prognosis in the TCGA dataset (Table 4). The prognosis of *SOX2*^High^ was good regardless of whether a history of RT was present (Figure S5), and the effect of RT was not significant when classified as *SOX2* expression (Figure S6). Subgroup analysis of more than two factors was difficult to perform due to the small number of patients and unequal deviations in the status of HPV and *SOX2* expression in the TCGA-HNSC dataset.

**Figure 7:**
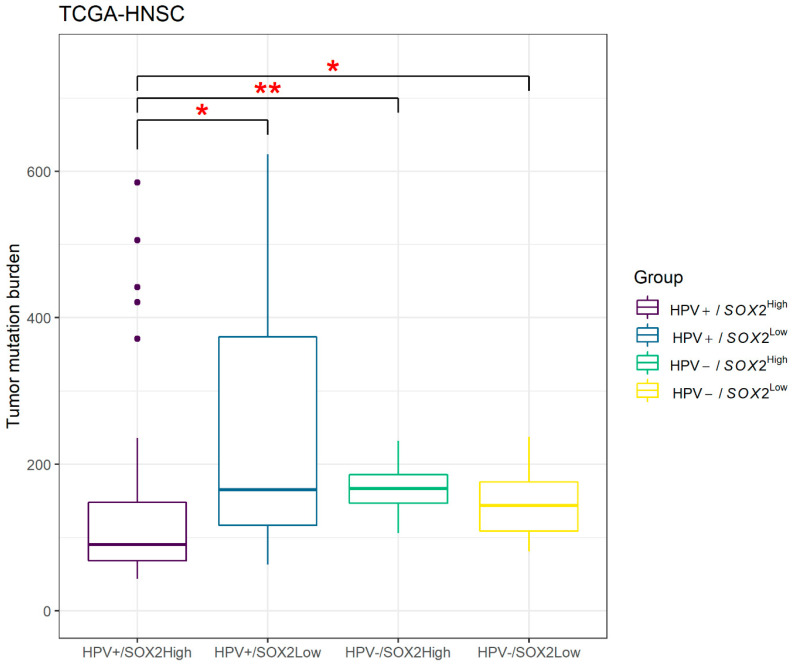
HPV+/*SOX2*^High^ scored significantly lower tumor mutation burden (** *p* < 0.01; * *p* < 0.05).

TMB was calculated in groups classified by HPV+/− and *SOX2*^High/Low^ (Figure 7). The TMB score of HPV+/*SOX2*^High^ (median [IQR]: 90.5 [68.4–147.9]) was the lowest among the four groups at a significant level; TMB of HPV+/*SOX2*^Low^ (median [IQR]: 165.5 [116.6–373.9], *p* = 0.042). HPV−/*SOX2*^High^ (median [IQR]: 166.8 [146.8–185.5], *p* = 0.005) and HPV−/*SOX2*^Low^ (median [IQR]: 143.8 [108.9–175.5], *p* = 0.025). However, there was no significant difference between *SOX2*^High^ and *SOX2*^Low^ in HPV− groups (*p* = 0.279).

From the immune landscape of OPSCC in the TCGA-HNSC dataset (Figure S7), HPV+ cancers had a significantly higher rate of immune infiltration than HPV− cancers (83.7% vs. 16.3%, *p* = 0.001). Three markers, PD-1, PD-L1 and CTLA-4, related to immune therapy were not significantly different according to SOX2 expression in either HPV+ or HPV− oropharyngeal cancers (all *p* > 0.05) (Figure S8).

## 4. Discussion

Over the past decades, the overall incidence of HNSCC has been decreasing in the United States, Europe and Australia, while the incidence of OPSCC has been increasing [28]. Infection with high-risk HPV, especially type 16, as a pathogen of HNSCC arising from the oropharynx has been implicated in this trend [29]. Although HNSCC has a high incidence of therapy-resistant local and regional recurrence and distant metastases [30], HPV+ OPSCC is regarded to have a better prognosis, better response to chemotherapy and radiotherapy and distinct biology from HPV- OPSCC [28,31]. However, to the best of our knowledge, there is no robust biomarker that can molecularly subclassify OPSCC after p16.

SOX2 has been reported to be related to HPV infection. HPV infection drives switches in SOX2 expression in the transformation zone in the uterine cervix [32], and SOX2 locus amplification was associated with HPV ISH positivity in vulvar carcinoma [33]. Furthermore, SOX2 was revealed to be a regulator of HPV16 at the transcriptional level [34]. Therefore, the purpose of the study was to analyze the implication of SOX2 in oropharyngeal cancer as the next available marker following p16 and to validate our results in the TCGA-HNSC dataset.

Even in the genomic era, IHC is still the most important tool because the method is quick, widely available, technically less challenging and cost-effective [35,36]. Here, SOX2 was quantified using IHC and stratified with p16 to confirm that the prognosis was significantly divided. This finding was confirmed in the TCGA-HNSC dataset using the *SOX2* mRNA expression level. This implies that SOX2, either by IHC scoring or measuring mRNA expression level, may be a potential prognostic marker for p16+ OPSCC. These results are in contrast to the known role of SOX2 because SOX2 is known to play an important role in maintaining the stemness of pluripotent stem cells, and many studies have reported that SOX2 is involved in cancer stem cell regulation [37]. A migrative, invasive and metastatic characteristic of tumors is the epithelial–mesenchymal transition (EMT), and SOX2 is well known to promote EMT in various cancers [6,37]. Nevertheless, whether the expression of SOX2 correlates with prognosis has not yet been determined, and the results vary from organ to organ, even in the head and neck cancer subsites [7,8,9,16,17,18,19]. For example, two studies showed the opposite results in HPV− HNSCC [38,39]. On the other hand, a meta-analysis revealed that SOX2 has poor outcomes [40]; however, SOX2 activation was associated with improved outcomes in the TCGA-HNSC dataset [19]. Specifically, higher SOX2 expression was reported to have better prognosis in oral cavity cancer [17,18], and the opposite result was reported in laryngeal cancer [16]. In their series, Dogan et al. reported that SOX2 overexpression was related to poor outcome only in HPV-OPSCC [13]. These varying results may be attributed to differences in patient groups for each study. Another possible reason may be that the interplay between multiple molecular factors may affect the course and severity of cancer [37].

In this study, one of the mechanisms underlying the poor prognosis of lower SOX2 IHC scores was that lower SOX2 scores were associated with advanced T classification (pT3 and -4), which was similar to other studies on oral cavity cancer [17,18]. Fu et al. considered SOX2 to play an important role in the early stages of tumorigenesis and that it was an independent prognostic marker [18]. However, contrary results have been reported; SOX2 expression was associated with large tumors [41]. In contrast to T classification, our data showed that N classification was not significantly correlated with SOX2. Studies on oral squamous cell cancer showed that the presence of nodal metastasis and lower SOX2 expression were associated [17,42], but not OPSCC for the patients in our study or TCGA-HNSC dataset. This result may be due to the small number of study populations or the anatomical difference between the oral cavity and oropharynx, but this cannot be concluded.

The second possible mechanism for the poor prognosis of lower SOX2 levels may be recurrence. When looking closely at the pattern of recurrence, the analysis revealed that SOX2 had a significant effect on locoregional recurrence independent of p16 expression level but not distant metastasis. In OPSCC, the results are still controversial, but there are several studies on the recurrence pattern according to the positivity of HPV [43,44,45]. However, SOX2 has not been discussed as much as HPV status. The results of this study suggest the role of SOX2 as a potential marker for the prediction of progression patterns. However, as a single institutional study, caution needs to be taken when interpreting the results and more evidence is needed.

Since evidence for p16 has been established, p16+ oropharyngeal cancer is generally regarded to have a better prognosis than p16−. In this study, the trend that p16+ had a better prognosis regardless of SOX2 score and mRNA expression level was confirmed, but there was no significant difference in 5-year OS and recurrence between p16+/SOX2^Low^ and p16−/SOX2^High^ in the TMA cohort and between HPV+/*SOX2*^Low^ and HPV−/*SOX2*^High^ in the TCGA−HNSC dataset. These data further imply that the combination of these markers might help with more precise molecular typing for HNSCC, either in p16+ or p16−. Similar results have not yet been previously questioned or reported. Since the study population was not sufficient, further analysis to draw conclusions cannot be done, but this should be confirmed in the future.

Cancer stem cell features are generally associated with higher radioresistance [46]. SOX2 is a regulatory marker of cancer stem cells, and accordingly, the association between SOX2 and radiosensitivity has been widely discussed [19,47,48,49]. Here, SOX2 showed a significant difference in OS and recurrence in patients receiving PORT; the prognosis of SOX2^High^ was better in 5-year OS and recurrence. In the subgroup analysis by p16+/− with PORT+/−, however, these differences were not significant. Although not significant, it is noteworthy that the difference in prognosis between SOX2^High^ and SOX2^Low^ was more prominent in PORT+/p16− than PORT+/p16+. Because RT-related outcome was known to be worse in p16− oropharyngeal cancer than p16+ [50], SOX2 needs to be further investigated as a candidate prognosticator to precisely select RT as a treatment option, especially in p16− OPSCC. In the TCGA-HNSC dataset, the effect of RT and SOX2 on prognosis was not significant.

Recently, immunotherapy has been introduced in the treatment of head and neck cancers. As the oropharynx is an immune-privileged site, OPSCC can be a candidate for immunotherapy [51]. As TMB is one of the response predictors for immuno-oncologic (I-O) treatment [52,53], we traced the TMB in our four groups according to SOX2 and HPV status. Interestingly, HPV+/*SOX2*^High^, which showed the best prognosis, had a significantly lower TMB among groups (all *p* < 0.05). However, these groups with higher TMB and worse prognosis might be the groups for which I-O treatment would work. Moreover, HPV+/*SOX2*^High^ might not be a good target for I-O treatment in terms of low TMB. In this case, other predictors might be combined to properly predict the response. Future trials are warranted.

There are several limitations in the study. The TMA cohort consists of patients who underwent surgery as an initial treatment and the results cannot be applied to all OPSCC patients. In addition, due to the relatively small number of populations, a larger-scaled prospective study is necessary to ensure the results of the study. Finally, given that we did not detect HPV infection directly through ISH, there may be a limit to interpreting the results because p16 does not guarantee the presence of HPV infection. However, the significance of this study may remain, given that HPV positivity of OPSCC is determined by p16 in the latest cancer staging manual [22].

## 5. Conclusions

In p16+ OPSCC, the SOX2 IHC score can be used as a marker to predict overall survival and recurrence. From analysis of the TCGA-HNSC dataset, *SOX2* mRNA expression was also proven to be a biomarker to predict prognosis. However, the role of SOX2 has not yet been confirmed in p16- (HPV−) patients. T classification and the RT response are being considered as mechanisms for SOX2 to have different outcomes in p16+ OPSCC, however, further study is needed to prove.

## Figures and Tables

**Table 1:** Demographics for 111 oropharyngeal cancer patients. Three patients without p16 immunohistochemistry (IHC) scores were excluded from the subgroup analysis.

			Total (n = 111)	p16+ (n = 67)	p16− (n = 41)	*p*
**Age at operation (years)**	Avg. ± SD		59.5 ± 10.8	57.0 ± 9.3	64.3 ± 11.3	<0.001 *
Sex	Male		97 (87.4%)	57 (85.1%)	38 (92.7%)	0.363 ^†^
	Female		14 (12.6%)	10 (14.9%)	3 (7.3%)	
Smoking	Yes		85 (76.6%)	46 (68.7%)	37 (90.2%)	0.010 ^††^
	No		26 (23.4%)	21 (31.3%)	4 (9.8%)	
Subsite	Tonsil		89 (80.2%)	59 (88.1%)	27 (65.9%)	0.007 ^††^
	Base of tongue		13 (11.7%)	7 (10.4%)	6 (14.6%)	
	Soft palate		6 (5.4%)	1 (1.5%)	5 (12.2%)	
	Uvula		3 (2.7%)	0 (0.0%)	3 (7.3%)	
T classification	pT3 and -4		19 (17.1%)	6 (9.0%)	13 (31.7%)	0.003 ^††^
	pT1 and -2		92 (82.9%)	61 (91.0%)	28 (68.3%)	
Nodal status	pN+		68 (61.3%)	47 (70.1%)	20 (48.8%)	0.060 ^††^
	pN−		35 (31.5%)	18 (26.9%)	17 (41.5%)	
	pNx (cN0)		8 (7.2%)	2 (3.0%)	4 (9.8%)	
Postoperative radiotherapy	Yes		78 (70.5%)	55 (80.9%)	22 (53.7%)	0.003 ^††^
	No		33 (29.5%)	13 (19.1%)	19 (46.3%)	
Follow-up duration (year)	Median [IQR]		4.6 [2.3–6.8]	5.4 [3.5–7.3]	2.3 [1.1–4.1]	<0.001 ^§^
Recurrence	Recurred		28 (25.2%)	7 (10.4%)	20 (48.8%)	0.007 ^††^
	NED ≥ 3 years		80 (72.1%)	59 (88.1%)	19 (46.3%)	
	PD or f/u loss		3 (2.7%)	1 (1.5%)	2 (2.9%)	
Survival	NED		63 (56.8%)	51 (76.1%)	10 (24.4%)	
	AWD		6 (5.4%)	0 (0.0%)	5 (12.2%)	
	DOD		23 (20.8%)	7 (10.5%)	16 (3.9%)	
	DOC		17 (15.2%)	8 (11.9%)	9 (22.0%)	
	f/u loss		2 (1.3%)	1 (1.5%)	1 (2.5%)	
SOX2 IHC score	High	30	74 (66.7%)	47 (70.1%)	24 (58.5%)	0.157 ^††^
		25–29	6 (5.4%)	4 (6.0%)	2 (4.9%)	
	Low	20	15 (13.5%)	10 (14.9%)	5 (12.2%)	
		10	13 (11.7%)	4 (6.0%)	9 (22.0%)	
		<5	3 (2.7%)	2 (3.0%)	1 (2.4%)	

Avg., average; SD, standard deviation; IQR, interquartile range; NED, no evidence of disease; PD, progression disease; IHC, immunohistochemistry; * Welch Two-Sample t-test, ^†^ Fisher’s exact test, ^††^ Chi-square test, ^§^ Wilcox rank sum test.

**Table 2:** Relationship between SOX2 immunohistochemistry score and pathologic T classification (pT) according to p16+ and p16−.

	p16+ Oropharyngeal Cancer (n = 67)			p16− Oropharyngeal Cancer (n = 41)		
	pT1 and -2	pT3 and -4	*p* *	pT1 and -2	pT3 and -4	*p* *
SOX2^High^	50 (82.0%)	1 (16.7%)	0.001	20 (71.4%)	6 (46.2%)	0.169
SOX2^Low^	11 (18.0%)	5 (83.3%)		8 (28.6%)	7 (53.8%)	

* Fisher’s exact test.

**Table 3:** Risk factor analysis for 5-year overall survival and recurrence in tumor microarray cohort.

	5-Year Overall Survival		5-Year Recurrence	
	HR (95% CI)	*p* *	HR (95% CI)	*p* *
**Univariate Analysis**				
p16− (vs. p16+)	6.16 (2.83–13.4)	<0.001	6.79 (2.98–15.5)	<0.001
SOX2^Low^ (vs. SOX2^High^)	2.39 (1.19–4.81]	0.015	2.72 (1.32–5.59)	0.005
PORT− (vs. PORT+)	2.70 (1.34–5.41)	0.004	2.02 (0.96–4.24)	0.065
**Multivariate Analysis**				
p16− (vs. p16+)	5.87 (2.69–12.8)	<0.001	6.26 (2.73–14.4)	<0.001
SOX2^Low^ (vs. SOX2^High^)	1.99 (0.99–4.04)	0.054	2.33 (1.11–4.89)	0.025
PORT− (vs. PORT+)		>0.05		>0.05

HR, hazard ratio; CI, confidence interval; PORT, postoperative radiotherapy; * Cox proportional hazard ratio model.

**Table 4:** Risk factor analysis for 5-year overall survival and recurrence in The Cancer Genome Atlas Head–Neck Squamous Cell Carcinoma (TCGA-HNSC) dataset.

	5-Year Overall Survival		5-Year Recurrence	
	HR (95% CI)	*p* *	HR (95% CI)	*p* *
**Univariate Analysis**				
HPV− (vs. HPV+)	7.11 (2.45–20.6)	<0.001	5.97 (2.33–15.3)	<0.001
*SOX2*^Low^ (vs. *SOX2*^High^)	8.98 (3.08–26.2)	<0.001	2.82 (1.12–7.09)	0.028
RT− (vs. RT+)		>0.05		>0.05
**Multivariate Analysis**				
HPV − (vs. HPV +)	3.24 (0.96–10.9)	0.059	5.56 (1.95–15.9)	0.001
*SOX2*^Low^ (vs. *SOX2*^High^)	4.87 (1.44–16.5)	0.011	1.17 (0.43–3.22)	0.759

HR, hazard ratio; CI, confidence interval; RT, radiotherapy; * Cox proportional hazard ratio model.

## Supplementary Materials

The following are available online at https://www.mdpi.com/2079-7737/9/11/387/s1. Figure S1: The score distribution of p16 and SOX immunohistochemistry from the tissue microarray cohort, Figure S2: Overall survival and recurrence according to the SOX2 IHC score in the TMA, Figure S3: Effect of SOX2 on overall survival and recurrence according to the classification with postoperative radiotherapy, Figure S4: Overall survival and recurrence according to *SOX2* mRNA expression level in patients with oropharyngeal squamous cell carcinoma in TCGA, Figure S5: The survival rate and recurrence rate of patients who underwent radiotherapy (RT+) and the survival rate and recurrence rate of those who did not (RT−), Figure S6: The survival rate and recurrence rate of patients with higher *SOX2* mRNA expression levels (*SOX2*^High^) and survival rate and recurrence rate of *SOX2*^Low^, Figure S7: Immune landscape of oropharyngeal cancers in the TCGA-HNSC dataset, Figure S8: The mRNA expression levels of three immune therapy-related markers (*PD-1*, *PD-L1*, and *CTLA-4*), Table S1: Risk analysis according to the pattern of recurrence using p16 and SOX2 in TMA datasets, Table S2: Demographics for 79 oropharyngeal cancer patients in the TCGA dataset, Table S3: Cox regression hazard ratio of *SOX2* mRNA expression for 5-year overall survival and recurrence rate in TCGA-HNSC dataset.
