# Supplementary material for: Prognostic Implication of SOX2 Expression Associated with p16 in Oropharyngeal Cancer: A Study of Consecutive Tissue Microarrays and TCGA
Source: Biology (Basel). 2020 Nov 9;9(11):387. doi: 10.3390/biology9110387 (PMC7695281; doi:10.3390/biology9110387)
Supplement: Supplementary file 1 [file biology-09-00387-s001.pdf]

Supplementary materials

Prognostic Implication of SOX2 Expression Associated with p16 in Oropharyngeal Cancer: A Study of Consecutive Tissue Microarrays and TCGA

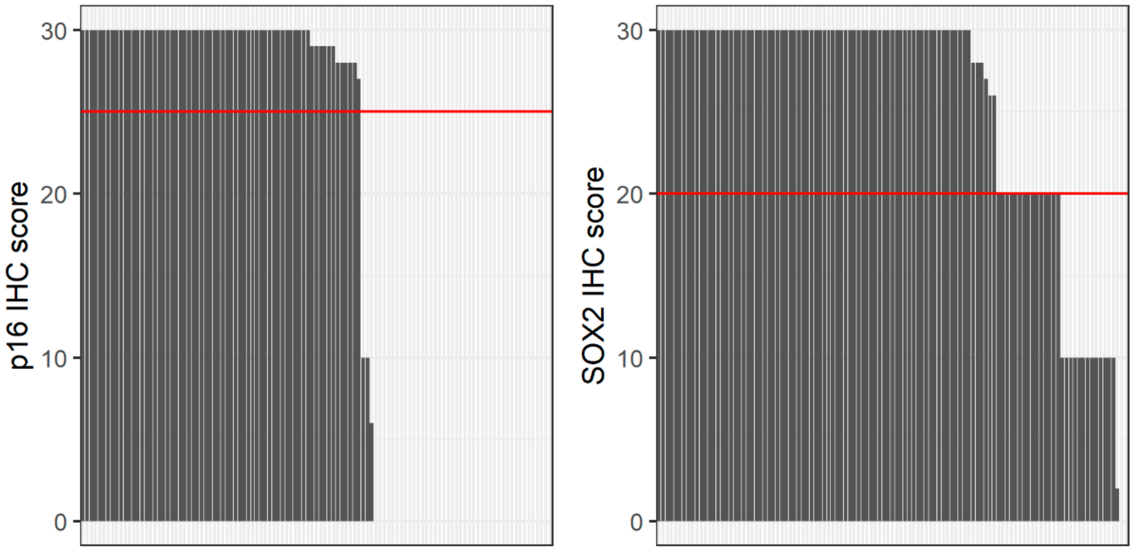

Figure S1. The score distribution of p16 and SOX immunohistochemistry from the tissue microarray cohort. The red line indicates the cutoff value for each marker (p16: 25, SOX2: 20). The SOX2 cutoff was determined using a time-dependent ROC curve. For p16, 67 patients (62.0%) were above the cutoff, and 41 (38.0%) were below the cutoff. For SOX2, 80 patients (72.1%) were above the cutoff, and 31 (27.9%) were below the cutoff.

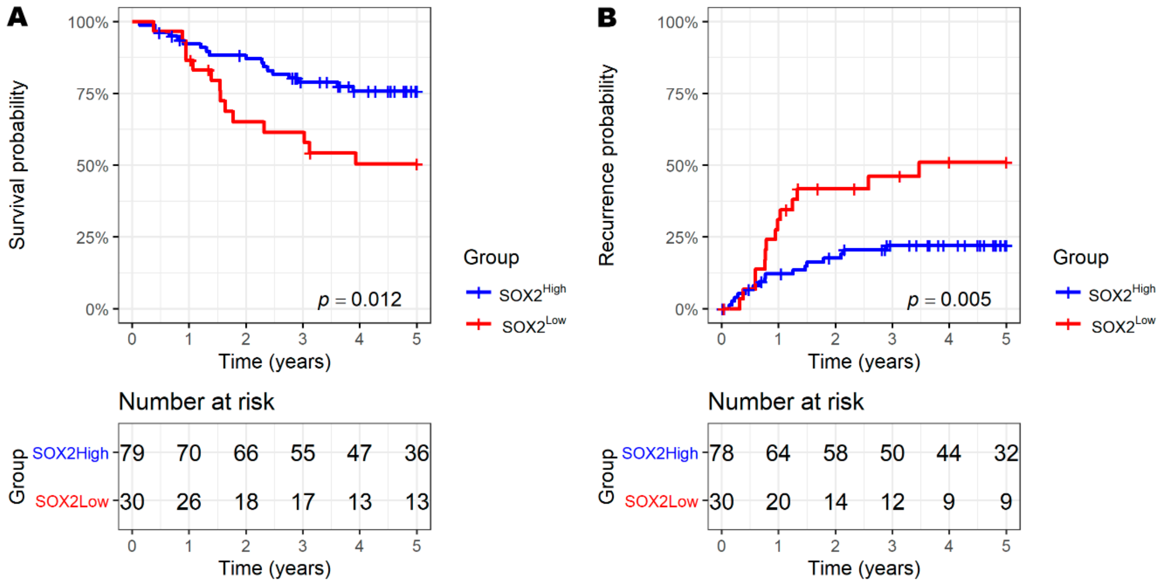

Figure S2. Overall survival (**A**) and recurrence (**B**) according to the SOX2 IHC score in the tissue microarray cohort.

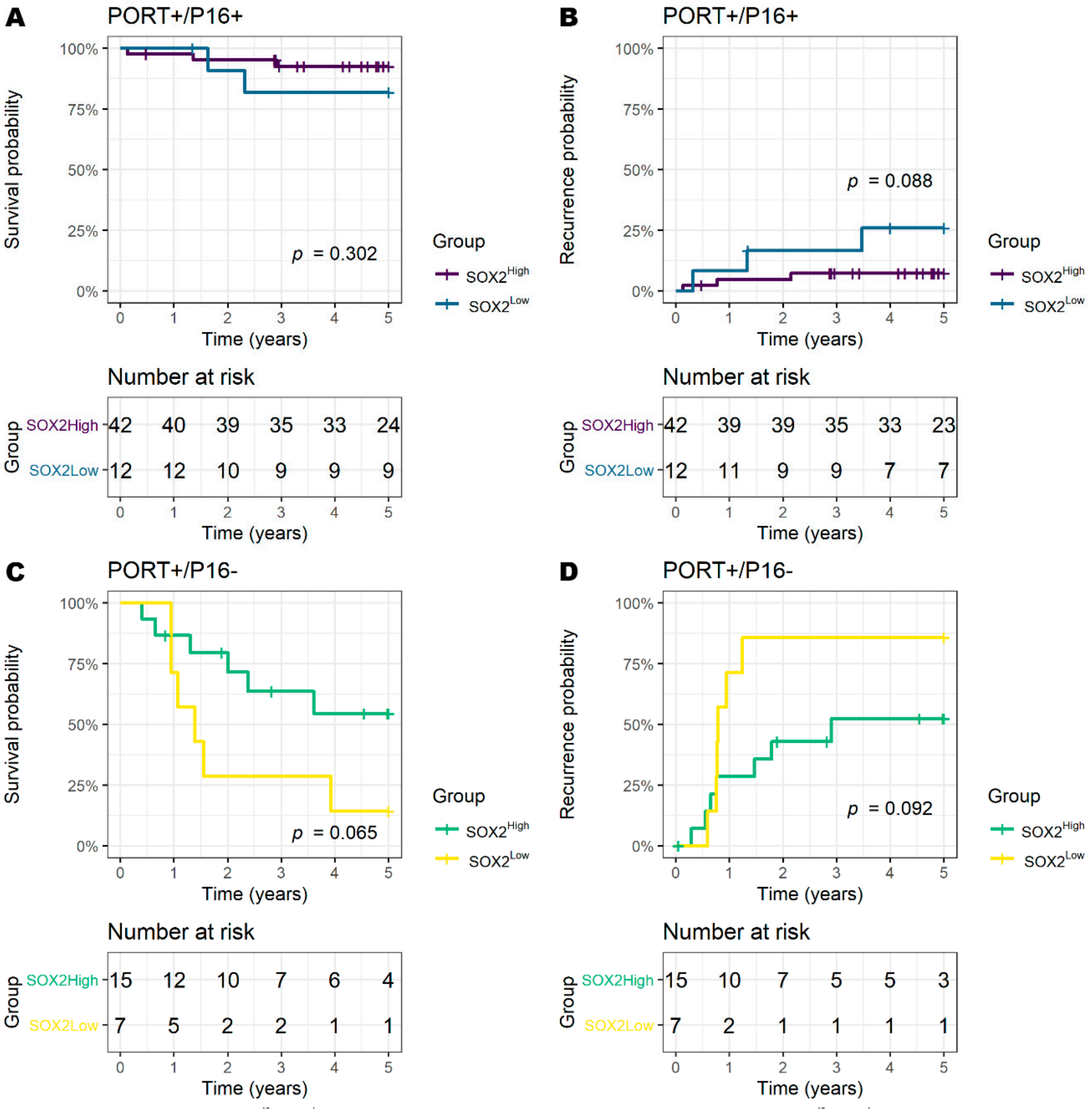

Figure S3. Effect of SOX2 on overall survival and recurrence according to the classification with postoperative radiotherapy (PORT) +/− and P16 +/− in the tissue microarray cohort. The prognosis of $SOX2^{High}$ tended to be good in all subgroups but not significant (all $p > 0.05$).

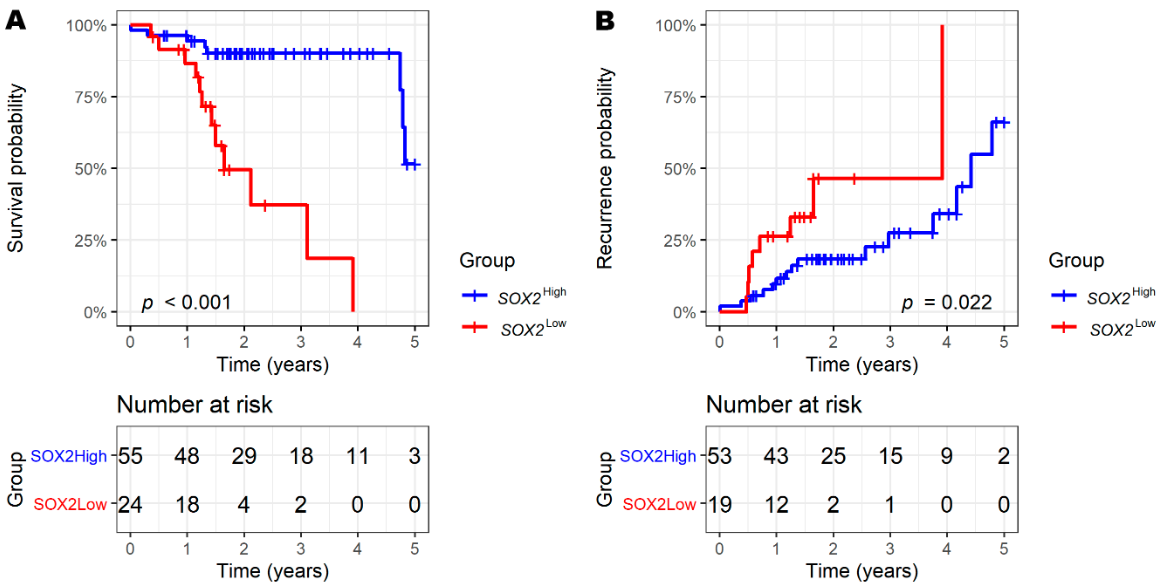

Figure S4. Overall survival (**A**) and recurrence (**B**) according to *SOX2* mRNA expression level in patients with oropharyngeal squamous cell carcinoma in the TCGA-HNSC dataset.

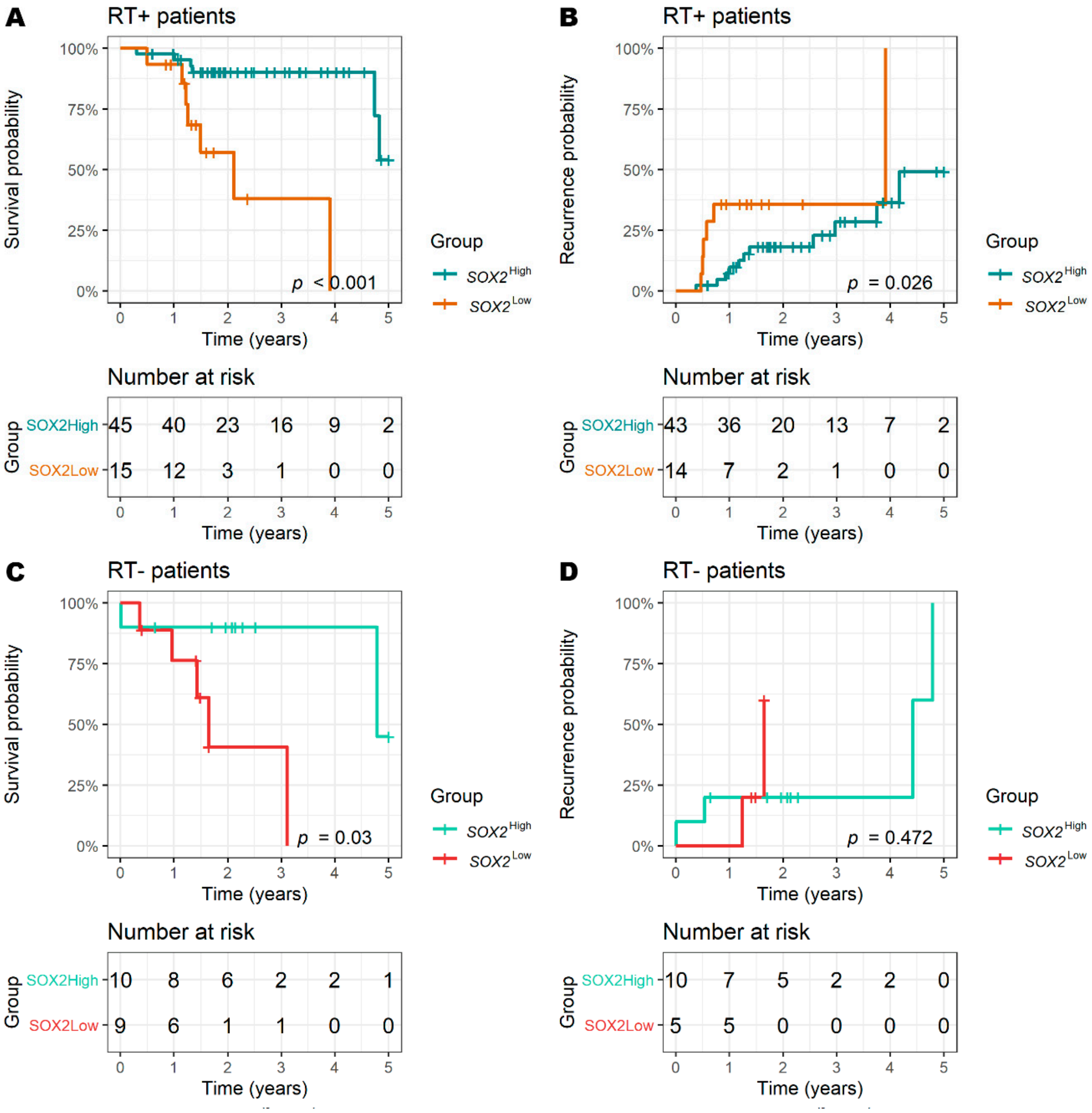

Figure S5. The survival rate (**A**) and recurrence rate (**B**) of patients who underwent radiotherapy (RT+) and the survival rate (**C**) and recurrence rate (**D**) of those who did not (RT−) in the TCGA-HNSC dataset. Overall survival of the *SOX2*^{High} group was significantly better regardless of RT (all $p < 0.05$).

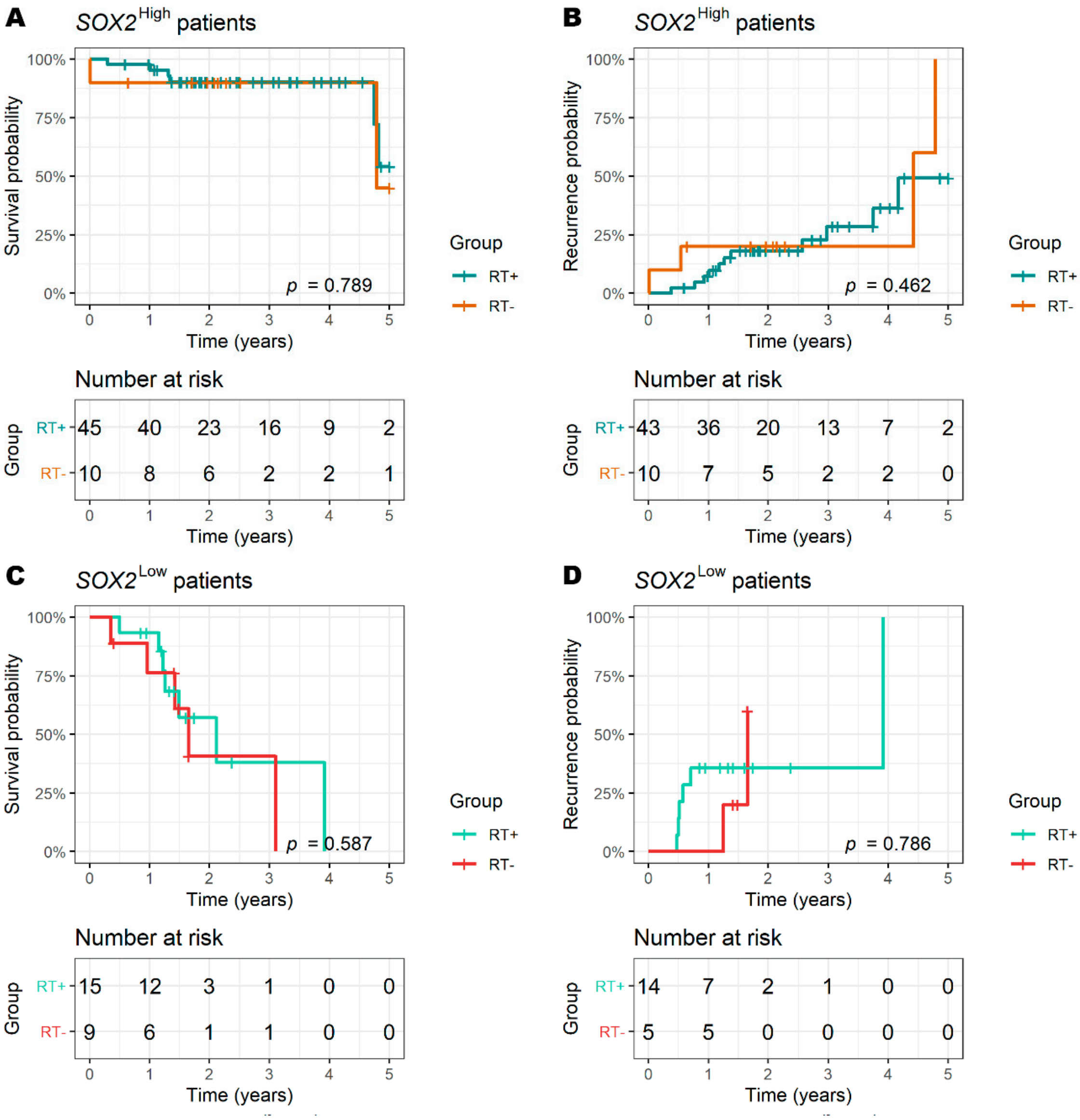

Figure S6. The survival rate (**A**) and recurrence rate (**B**) of patients with higher *SOX2* mRNA expression levels (*SOX2*^{High}) and survival rate (**C**) and recurrence rate (**D**) of *SOX2*^{Low} in the TCGA-HNSC dataset. Radiotherapy did not significantly affect prognosis in each group (all $p > 0.05$).

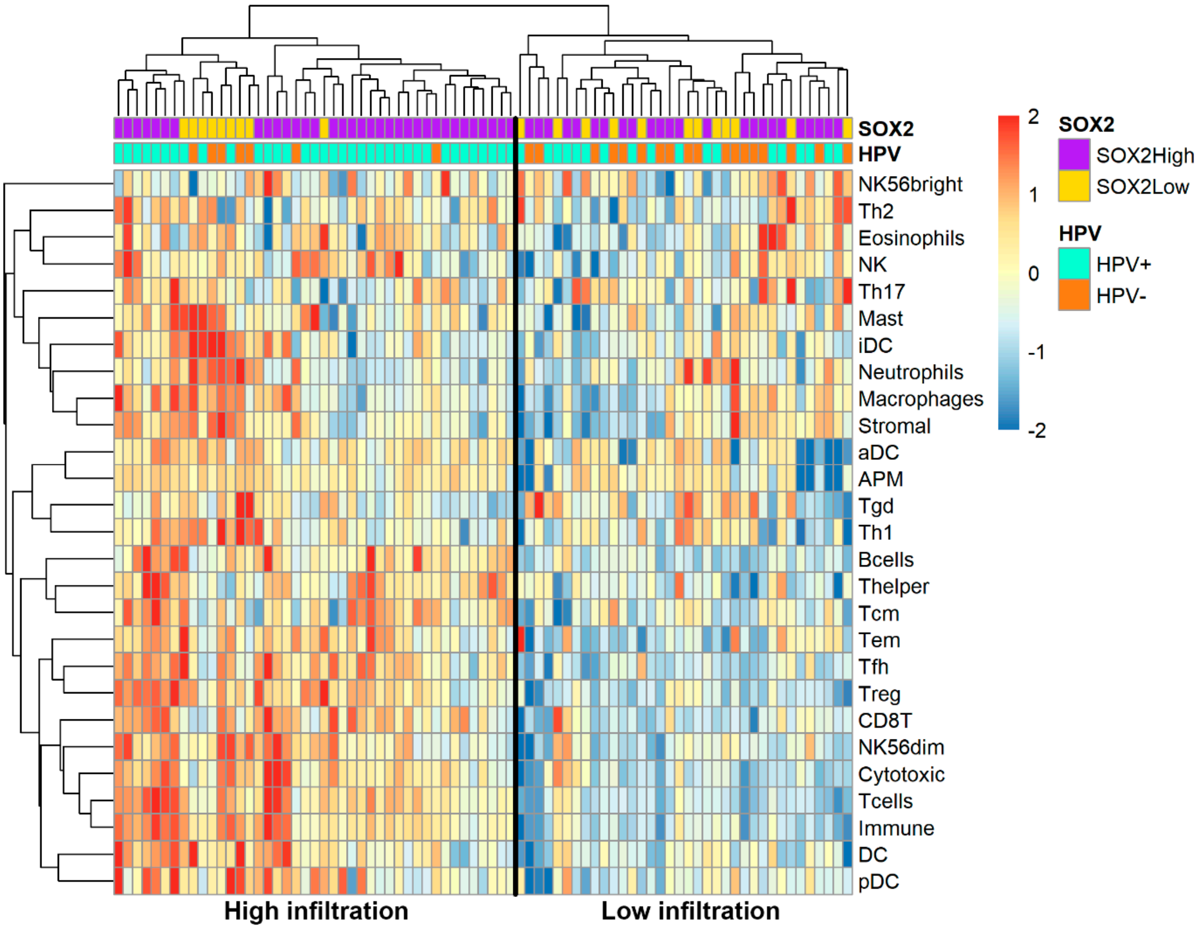

Figure S7. Immune landscape of oropharyngeal cancers in the TCGA-HNSC dataset. The distribution of *SOX2*^{High} and *SOX2*^{Low} was not significantly different regarding immune cell infiltration ($p = 0.214$). However, the distribution of HPV status was significantly different; HPV+ cancers belonged to the high infiltration group at a significantly higher rate (HPV+ vs HPV−: 83.7% vs 16.3% in high immune infiltration, 50% vs 50% in low immune infiltration, $p = 0.001$).

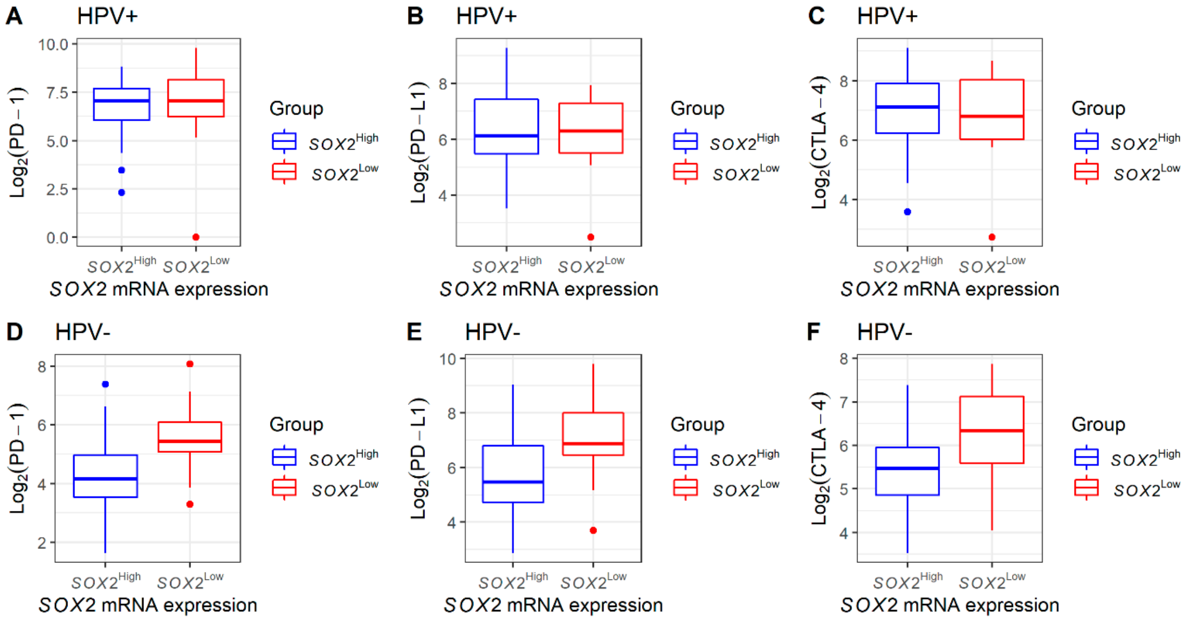

Figure S8. The mRNA expression levels of three immune therapy-related markers (*PD-1*, *PD-L1*, and *CTLA-4*) were not significantly different between *SOX2*High and *SOX2*Low in both HPV+ (**A**: *PD-1*, **B**: *PD-L1*, and **C**: *CTLA-4*) and HPV− (**D**: *PD-1*, **E**: *PD-L1*, and **F**: *CTLA-4*) oropharyngeal cancer in the TCGA-HNSC dataset (all $p > 0.05$).

Table S1. Risk analysis according to the pattern of recurrence using p16 and SOX2 in the tissue microarray cohort.

	Locoregional recurrence		Distant metastasis	
	**HR [95% CI]**	p^*	HR [95% CI]	p^*
**Univariate analysis**				
p16− (vs p16+)	6.32 [2.40–16.7]	<0.001	5.54 [1.37–22.5]	0.017
$SOX2^{Low}$ (vs $SOX2^{High}$)	3.12 [1.29–7.53]	0.011	2.55 [0.68–9.57]	0.165
**Multivariate analysis**				
p16− (vs p16+)	5.81 [2.19–15.4]	<0.001	5.18 [1.26–21.2]	0.022
$SOX2^{Low}$ (vs $SOX2^{High}$)	2.66 [1.09–6.47]	0.032	2.17 [0.57–8.27]	0.255

HR, hazard ratio; CI, confidence interval; * Cox proportional hazard ratio model.

Table S2. Demographics for 79 oropharyngeal cancer patients in the TCGA-HNSC dataset.

		**Total (n=79)**	**HPV+ (n=54)**	**HPV− (n=25)**	***p***
Age at operation (year)	Avg. ± SD	55.9 ± 9.3	54.6 ± 9.1	58.7 ± 9.3	0.075*
Sex	Male	68 (86.1%)	49 (90.7%)	19 (76.0%)	0.093†
	Female	11 (13.9%)	5 (9.3%)	6 (24.0%)	
Subsite	Tonsil	43 (54.4%)	36 (66.7%)	7 (28.0%)	<0.001††
	Base of tongue	27 (34.2%)	16 (29.6%)	11 (44.0%)	
	Other oropharynx	9 (11.4%)	2 (3.7%)	7 (28.0%)	
T classification	T3&4	32 (40.5%)	15 (27.7%)	17 (68.0%)	0.002††
	T1&2	47 (59.5%)	39 (72.2%)	8 (32.0%)	
Nodal status	N+	59 (74.7%)	43 (79.6%)	16 (64.0%)	0.227††
	N−	20 (25.3%)	11 (20.4%)	9 (36.0%)	
Radiotherapy	Yes	60 (75.9%)	40 (74.1%)	20 (80.0%)	0.772††
	No	19 (24.1%)	14 (25.9%)	5 (20.0%)	
Follow-up duration (year)	Median [IQR]	1.7 [1.3–3.0]	2.0 [1.4–3.3]	1.4 [1.2–2.1]	0.018§
Recurrence	Recurred	21 (26.6%)	9 (16.7%)	12 (48.0%)	
	NED≥3 years	11 (13.9%)	10 (18.5%)	1 (4.0%)	
	Follow-up < 3 years or SD, PD	46 (59.5%)	35 (64.8%)	12 (48.0%)	
Survival	NED	11 (13.9%)	10 (18.5%)	1 (4.0%)	
	AWD	5 (6.3%)	5 (9.3%)	0 (0.0%)	
	DOD	9 (11.4%)	6 (11.1%)	3 (12.0%)	
	DOC	5 (6.4%)	1 (1.9%)	4 (16.0%)	
	NED & AWD but follow-up < 3 years	49 (62.0%)	32 (59.2%)	17 (68.0%)	
SOX2 mRNA expression	Median [IQR]	0.13 [−0.43–0.65]	0.35 [−0.17–0.71]	−0.59 [−1.31–0.38]	0.005§
SOX2 mRNA expression (cut-off: −0.3020)	High	55 (69.6%)	44 (81.5%)	11 (44.0%)	0.001††
	Low	24 (30.4%)	10 (18.5%)	14 (56.0%)	

Avg., average; SD, standard deviation; IQR, interquartile range; NED, no evidence of disease; PD, progression disease; IHC, immunohistochemistry; * Welch Two Sample t-test, † Fisher's Exact test, †† Chi-square test, § Wilcox rank sum test

Table S3. Cox regression hazard ratio of *SOX2* mRNA expression for 5-year overall survival and recurrence rate in the TCGA-HNSC dataset.

HPV status			5-year overall survival		5-year recurrence	
			**HR [95% CI]**	***p****	**HR [95% CI]**	***p****
**HPV+**	*SOX2* (continuous)	per minus 1 †	4.24 [1.18–15.3]	0.027	1.41 [0.40–4.94]	0.589
	SOX2 (category)	Low (vs High) ††	2.34 [1.66–65.6]	0.012	–	0.999¶
**HPV−**	*SOX2* (continuous)	per minus 1 †	1.48 [0.93–2.35]	0.097	1.05 [0.63–1.76]	0.849
	SOX2 (category)	Low (vs High) ††	2.52 [0.68–9.35]	0.168	1.48 [0.48–4.55]	0.491

HR, hazard ratio; CI, confidence interval; * Cox proportional hazard ratio model, † Log-scaled and z-transformed mRNA expression value, †† Cut-off values was 0.0302, calculated from survival ROC method. Statistical calculation was not performed because there were no patients with recurrence in the HPV+ and Low *SOX2* groups.
